# The effect comparison of ILM flap and traditional ILM peeling in iMH

**DOI:** 10.3389/fmed.2023.1103593

**Published:** 2023-02-09

**Authors:** Yiqi Chen, Yijun Xu, Xin Ye, Jiafeng Yu, Chenxi Wang, Zhengxi Zhang, Jianbo Mao, Lijun Shen

**Affiliations:** ^1^Department of Ophthalmology, Zhejiang Provincial People’s Hospital, Hangzhou, Zhejiang, China; ^2^School of Ophthalmology and Optometry, Wenzhou Medical University, Wenzhou, Zhejiang, China; ^3^Daxing Teaching Hospital Affiliated to Capital Medical University, Beijing, China

**Keywords:** idiopathic macular hole, internal limiting membrane, the inverted ILM flap treatment, visual function, optical coherence tomography

## Abstract

**Purpose:**

To compare the changes in anatomical structure and visual function after idiopathic macular hole (iMH) treatment with internal limiting membrane (ILM) peeling and inverted ILM flap and determine the value of the inverted ILM flap for the treatment of iMH.

**Methods:**

Forty-nine patients with iMH (49 eyes) were included in this study and followed up for 1 year (12 months) after treatment with inverted ILM flap and ILM peeling respectively. The main foveal parameters assessed included the preoperative minimum diameter (MD), intraoperative residual fragments, and postoperative ELM reconstruction. Visual function was assessed using best-corrected visual acuity.

**Results:**

The hole closure rate was 100% for 49 patients; 15 patients were treated with the inverted ILM flap, and 34 patients underwent ILM peeling. There were no differences between the postoperative best-corrected visual acuities and the rates of ELM reconstruction for the flap and peeling groups with different MDs. In the flap group, ELM reconstruction was associated with the preoperative MD, presence of an ILM flap, and hyperreflective changes in the inner retina 1 month after surgery. In the peeling group, ELM reconstruction was associated with the preoperative MD, intraoperative residual fragments at the hole edge, and hyperreflective changes in the inner retina.

**Conclusion:**

The inverted ILM flap and the ILM Peeling were both able to obtain high closure rate. However, the inverted ILM flap showed no obvious advantages related to anatomical morphology and visual function over ILM peeling.

## Introduction

Idiopathic macular holes (iMHs) are full-thickness anatomical defects at the fovea caused by traction of the vitreous and internal limiting membrane (ILM). The conventional treatment for iMH is pars plana vitrectomy (PPV) combined with ILM peeling to release traction (1), and postoperative macular hole closure rates of up to 100% have been reported (2–4). However, for the patients with the refractory iMHs (the large iMH and myopic MHs [with or without retinal detachment]) the postoperative closure rate after ILM peeling is only 61–100%, which is relatively low (5, 6). Michalewska (7) proposed the inverted ILM flap in 2014 to significantly improve the postoperative closure rate of refractory macular holes. A study of large iMHs suggested that the macula hole closure rate increased to more than 90% with ILM flap surgery (8–10), which markedly reduced the risk of postoperative recurrence of macular holes.

Subsequently, several studies have investigated the visual function and anatomical changes in patients with large iMHs after the inverted ILM flap. Rizzo et al. (11) found that the inverted ILM flap for large iMHs results in better visual outcomes. A systematic review meta-analysis recommended the inverted ILM flap for large iMHs (12, 13). However, the meta-analysis had limited reliability, and the postoperative ELM reconstruction status was not evaluated. Some previous studies have also reported that the inverted ILM flap improves the closure rate of large iMHs but not visual function compared with ILM peeling (14, 15). Yan et al. found no difference between the closure rates after the inverted ILM flap and ILM peeling for large iMH (16). However, these studies had fewer cases and shorter follow-ups. Therefore, the value of the inverted ILM flap for the treatment of macula holes needs to be established.

Therefore, the purpose of this study was to compare the changes in anatomical structure and visual function after ILM peeling and the inverted ILM flap for the treatment of iMH with different minimum diameters (MDs) to establish the value of the inverted ILM flap for iMH.

## Materials and methods

This was a retrospective study that included 49 eyes of 49 people who visited the Department of Retina Center, Affiliated Eye Hospital of Wenzhou Medical University, Hangzhou, Zhejiang Province, China, from January 2018 to January 2020. The participants had idiopathic full-thickness macular holes. The exclusion criteria included secondary MHs, such as those caused by trauma, high myopia (axial length [AL] ≥ 26.00 mm or refractive error of ≥ –6.00 D); MH with retinal detachment; other retinal vascular diseases, such as diabetic retinopathy, retinal vascular occlusion, and retinal inflammatory diseases; last follow-up visit within 12 months; and history of intraocular surgery.

This study was approved by the Institutional Review Board of Wenzhou Medical University, and the procedure complied with the principles of the Declaration of Helsinki. All patients signed an informed consent form. At the least, the informed consent form requested patient permission to allow researchers to collect information during clinical visits and use it for scientific research and article publication.

All patients underwent tests for BCVA (measured with the Snellen visual acuity chart and converted to logMAR for recording) and refractive error (spherical equivalent), intraocular lens assessments with IOL-Master 700, spectral domain optical coherence tomography (OCT) (SD-OCT), and intraoperative OCT (iOCT). All eyes were diagnosed with full-thickness macular holes by SD-OCT, and all patients were assessed at baseline and followed up 1, 3, 6, and 12 months after surgery.

**FIGURE 1:**
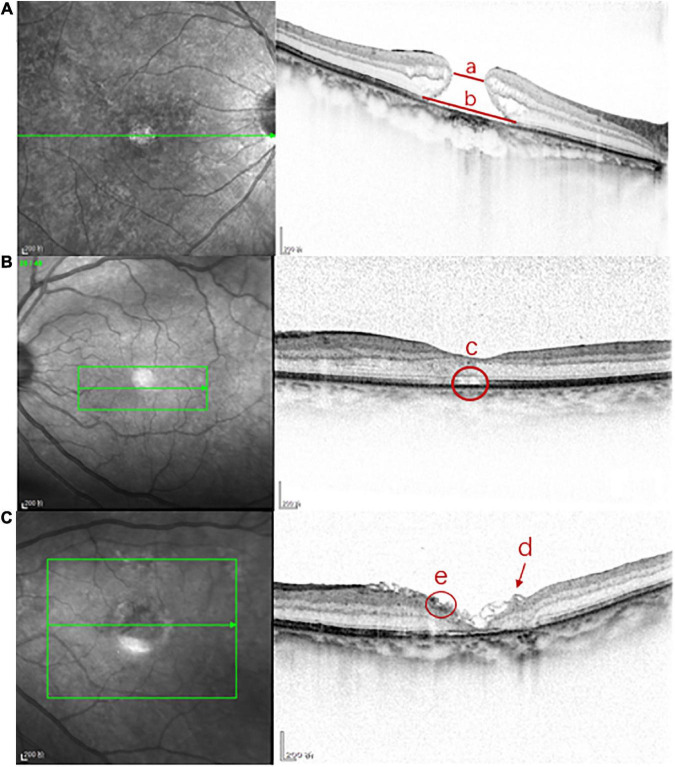
The foveal microstructures in iMH were evaluated by SD-OCT. **(A)** Line a is the preoperative minimum diameter of iMH, line b is the preoperative length of ELM defect of iMH; **(B)** c is the postoperative subretinal cavity; **(C)** the arrow d is the postoperative ILM flap in FLAP group, the circle e is the postoperative hyperreflective change of the inner retina.

The foveal microstructures in the eyes with iMHs were evaluated using SD-OCT (Heidelberg, Spectralis OCT). High-density central horizontal scanning in EDI mode was adopted and used for the central 5.8 × 5.8 mm with a 120-μm line spacing. The preoperative MD and the size of the external limiting membrane (ELM) defect were measured by software embedded in the OCT scanner (Figure 1). The postoperative foveal microstructures and characteristics included the macular hole reconstruction layer, hyperreflective changes in the inner retina, ILM flap, and subretinal cavity (Figure 1). All measurements were conducted by one professional technician; three measurements were taken and an average was obtained.

**FIGURE 2:**
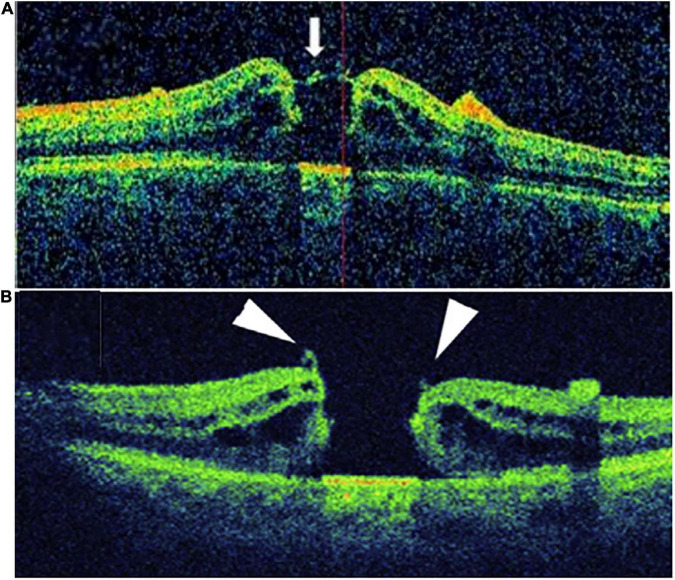
Patients with RF were divided into two groups according to the location of the RF: **(A)** RF at the hole edge group (RFHE), **(B)** RF not at the hole edge group (non- RFHE).

The intraoperative foveal microstructures were evaluated using the Optovue iVue OCT System (Optovue, Inc., Fremont, CA, USA). The scanning speed was 26,000 times/min, and the wavelength was 830 nm. Images acquired before and after ILM peeling were analyzed for qualitative changes. iOCT was mainly used to observe the residual fragments (RFs) of the macular hole after ILM peeling, which were categorized into RFs at the hole edge (RFHEs) and RFs outside the hole edge (Figure 2).

### Surgical technique

All patients were treated through 23-gauge PPV with ILM peeling by one surgeon (Shen). All patients also underwent cataract surgery. Indocyanine green staining (0.02 ml [0.025 mg/ml]) for the ILM was performed after routine vitrectomy.

#### ILM peeling (peeling group)

After indocyanine green staining, the ILM in the macular area was removed (range: 2 to 4 PD). The peeling was initiated from the temporal area of the macula. The ILM was peeled off 360 degrees centripetally and over the MH edge.

#### The inverted ILM flap (flap group)

During the removal of the ILM, the ILM at the temporal hole margin was retained. The hole margin tension was released, and the flap was inverted to cover the hole from the temporal to the nasal side.

Air-fluid exchange was performed by disinfecting the air at the end of the surgery, and all patients were required to maintain a face-down position for 7 days postoperatively.

### Statistical analysis

SPSS software was used for all statistical analyses (version 21; SPSS, Inc., Chicago, IL). *P*-values less than 0.05 were considered significant. Fisher’s exact and Chi-squared tests were used to compare the categorical data. The continuous variables are expressed as the mean ± standard deviation (range). The continuous data were evaluated for normal distribution using the Shapiro - Wilk test. The *t*-test was used to analyze the data conforming to the normal distribution, and the Mann-Whitney U test was used to analyze non-normally distributed data. Pearson’s or Spearman’s analysis was used for correlation analysis. The ROC curve was used to evaluate the MDs for the postoperative reconstruction of ELM, and the critical value of the MD was calculated. Based on the critical value, the patients were divided into two subgroups for comparison.

## Results

The ages of the 49 patients (49 eyes) with iMH ranged from 51 to 75 years (63.09 ± 5.96) (Table 1). Twelve of the participants were males. There were 15 patients in the flap group and 34 patients in the peeling group. The hole closure rate was 100% at 12 months postoperatively, and the ELM closure rate was 67%; the ELM closure rate was 60% for the flap group and 71% for the peeling group. There were significant differences between the preoperative and postoperative 12-month BCVAs of the peeling and flap groups (0.99 ± 0.44 vs. 0.46 ± 0.43 [*P* = 0.023] and 0.95 ± 0.39 vs. 0.54 ± 0.54, [*P* = 0.000], paired *t*-test).

**TABLE 1:** The common material for 49 iMH patients.

	Preoperative	Postoperative 1-month	Postoperative 3-month	Postoperative 6-month	Postoperative 12-month
Male/Female	12/37	–	–	–	–
Age	63.09 ± 5.96	–	–	–	–
AL	23.44 ± 0.89	–	–	–	–
MD	519.46 ± 179.31	–	–	–	–
BCVA	0.98 ± 0.42	0.72 ± 0.44	0.50 ± 0.41	0.48 ± 0.43	0.48 ± 0.46*
Hole closure rate	–	100%	100%	100	100
ELM closure rate	–	34.04%	54.76%	62.07%	67.35%

*There was a statistically significant difference between the preoperative and postoperative 12-month BCVAs (*P* = 0.000).

### Comparison of the flap and peeling groups

The preoperative and postoperative anatomical morphology and visual function of the flap and peeling groups were compared (Table 2). There were no significant differences between the ALs, MDs, preoperative BCVAs, and postoperative ELM reconstruction rates and BCVAs of the groups (*P* > 0.05, independent sample *t*-test).

**TABLE 2:** The preoperative and postoperative anatomical morphology and visual function of the flap and peeling groups***.

	Flap (15)	Peeling (34)	*P*
AL	23.41 ± 0.94	23.70 ± 1.70	0.551
Preoperative MD	524.27 ± 193.04	517.27 ± 175.80	0.902
Preoperative length of ELM defect	1,269.73 ± 502.67	1280.48 ± 611.18	0.625
Preoperative BCVA	0.95 ± 0.39	0.99 ± 0.44	0.991
**Postoperative 1-month**			
BCVA	0.74 ± 0.48	0.71 ± 0.43	0.832
ILM flap	10/5	0/34	0.000
Hyperreflective change of the inner retina	7/8	18/14	0.755
ELM reconstruction	5/10	11/21	1.000
Subretinal cavity	7/8	15/17	1.000
**Postoperative 12-month**			
BCVA	0.54 ± 0.54*	0.46 ± 0.43**	0.884
Hyperreflective change of the inner retina	3/12	11/23	0.502
ELM reconstruction	9/6	24/10	0.520
EZ reconstruction	1/14	7/27	0.406
Subretinal cavity	6/9	18/16	0.538

*There was a statistically significant difference between the preoperative and postoperative 12-month BCVAs (*P* = 0.000).

**There was a statistically significant difference between the preoperative and postoperative 12-month BCVAs (*P* = 0.023).

***The power of the chi-squared test is 93.8%.

**FIGURE 3:**
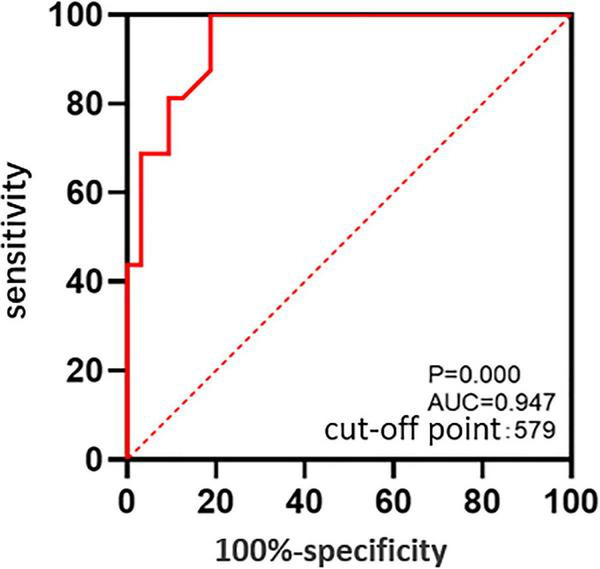
The ROC curve: The predictive value of the MD for postoperative reconstruction states of ELM.

The ROC curve suggested that the MD was predictive of postoperative ELM reconstruction (*P* = 0.000, AUC = 0.947), and the cut-off point of MD was 579 μm (Figure 3). The two groups were subdivided into four according to the MD. The preoperative and postoperative anatomical changes and visual function of the flap and peeling groups were compared for MD of > 579 μm (Table 3) and ≤ 579 μm (Table 4), respectively, but there were no statistical differences (*P* > 0.05, independent sample t-test and Shapiro - Wilk tests).

**TABLE 3:** Comparison of the preoperative and postoperative anatomical structures and visual function of the flap and peeling groups for MDs of > 579 μm*.

	Flap (8)	Peeling (15)	*P*
AL	23.34 ± 1.16	23.54 ± 9.24	0.690
Preoperative MD	671.63 ± 43.70	642.56 ± 94.38	0.126
Preoperative length of ELM defect	1,572.25 ± 196.56	1551.22 ± 458.19	0.404
Preoperative BCVA	1.21 ± 0.28	1.20 ± 0.44	0.601
Postoperative 1-month BCVA	0.99 ± 0.52	0.83 ± 0.47	0.548
Postoperative 12-month BCVA	0.88 ± 0.51	0.61 ± 0.49	0.102
**Postoperative 1-month**			
Hyperreflective change of the inner retina	7/1	3/12	0.006
ELM reconstruction	0/8	1/14	1.000
subretinal cavity	2/6	2/13	0.589
**Postoperative 12-month**			
Hyperreflective change of the inner retina	3/5	10/5	0.221
ELM reconstruction	2/6	5/10	1.000
EZ reconstruction	0/8	2/13	0.526
subretinal cavity	3/5	3/12	0.621

*The power of the chi-squared test is 66.9%.

**TABLE 4:** Comparison of the preoperative and postoperative anatomical structures and visual function of the flap and peeling groups for MDs of ≤ 579 μm*.

	Flap (7)	Peeling (19)	*P*
AL	23.51 ± 0.64	23.91 ± 2.41	0.509
Preoperative MD	355.86 ± 150.60	366.93 ± 124.22	0.751
Preoperative length of ELM defect	924.00 ± 532.01	955.60 ± 625.74	0.888
Preoperative BCVA	0.65 ± 0.27	0.70 ± 0.25	0.469
Postoperative 1-month BCVA	0.45 ± 0.18	0.54 ± 0.29	0.631
Postoperative 12-month BCVA	0.14 ± 0.16	0.26 ± 0.19	0.208
**Postoperative 1-month**			
Hyperreflective change of the inner retina	0/7	6/12	0.137
ELM reconstruction	5/2	10/8	0.659
subretinal cavity	5/2	12/6	1.000
**Postoperative 12-month**			
Hyperreflective change of the inner retina	0/7	1/18	1.000
ELM reconstruction	7/0	19/0	1.000
EZ reconstruction	1/6	5/14	1.000
subretinal cavity	3/4	15/4	0.149

*The power of the chi-squared test is 72.2%.

### Flap group: Differences in anatomical structure and visual function in different postoperative ELM reconstruction groups

The Flap group was divided into two based on the postoperative reconstruction of the ELM (Table 5, independent sample t-test and Mann–Whitney U tests); 9 patients were included in the ELM group. There were significant differences in the MD and preoperative BCVA between the two groups (*P* = 0.010, *P* = 0.010). The hyperreflective changes of the inner retina and ILM flap of the non-ELM group were significantly more than those of the ELM group 1 month after surgery (*P* = 0.001 and 0.044, respectively); the subretinal cavities were also significantly less in the non-ELM than in the ELM group (*P* = 0.007). The BCVAs were significantly better for the ELM group than for the non-ELM group (*P* = 0.013), and the hyperreflective changes of the inner retina in the non-ELM group were significantly more than those in the ELM group (*P* = 0.044).

**TABLE 5:** Comparison of the preoperative and postoperative anatomical structures and visual function of the ELM and non-ELM subgroups of the flap group*.

	Flap		*P*
	ELM (9)	Non-ELM (6)	
AL	23.58 ± 0.71	23.18 ± 1.21	0.519
Preoperative MD	425.22 ± 190.00	672.83 ± 49.35	0.010
Preoperative length of ELM defect	1,063.67 ± 543.55	1,578.83 ± 209.05	0.077
Preoperative BCVA	0.74 ± 0.32	1.27 ± 0.26	0.008
Postoperative 1-month BCVA	0.44 ± 0.16	1.11 ± 0.48	0.009
Postoperative 12-month BCVA	0.26 ± 0.32	0.96 ± 0.54	0.013
**Postoperative 1-month**			
ILM flap	4/5	6/0	0.044
hyperreflective change of the inner retina	1/8	6/0	0.001
ELM reconstruction	5/4	0/6	0.044
subretinal cavity	7/2	0/6	0.007
**Postoperative 12-month**			
Hyperreflective change of the inner retina	0/9	3/3	0.044
ELM reconstruction	9/0	0/6	0.000
subretinal cavity	5/4	1/5	0.287

* The power of the chi-squared test is 50%.

### Flap group: Correlation analysis of the 12-month ELM reconstruction

**FIGURE 4:**
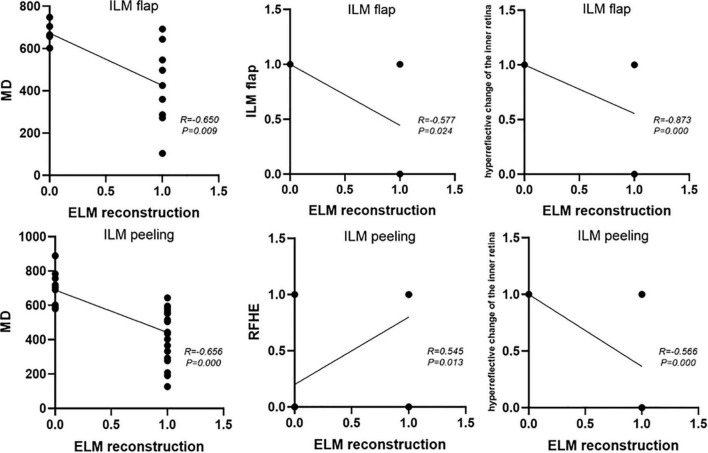
The factors affecting postoperative ELM reconstruction in the flap group and the peeling group.

ELM reconstruction 12 months after surgery was closely related to the preoperative MD (*P* = 0.009; *R* = −0.650), preoperative BCVA (*P* = 0.005, *R* = −0.684), presence of the ILM flap 1 month after surgery (*P* = 0.024, *R* = −0.577), and hyperreflective changes in the inner retina (*P* = 0.000, *R* = −0.873), respectively (Figure 4).

### Peeling group: Differences in anatomical structure and visual function in different postoperative ELM reconstruction groups

The peeling group was divided into two according to the postoperative reconstruction of the ELM (Table 6, independent sample t-test and Shapiro - Wilk); 24 patients were included in the ELM group. There were significant differences in the preoperative MD, size of the ELM defect, and BCVA of the two groups (*P* = 0.000, *P* = 0.000, *P* = 0.006). iOCT showed significantly more RFHEs in the ELM group than in the non-ELM group (*P* = 0.031). After 1 month postoperatively, the hyperreflective changes in the inner retina in the non-ELM group were significantly more than those in the ELM group (*P* = 0.001), and the subretinal cavities were significantly less in the non-ELM group than in the ELM group (*P* = 0.001). BCVA was significantly better for the ELM group than for the non-ELM group 12 months postoperatively (*P* = 0.000), and the hyperreflective changes in the inner retina of the non-ELM group were significantly more than those of the ELM group (*P* = 0.000).

**TABLE 6:** Comparison of the preoperative and postoperative anatomical structures and visual function of the ELM and non-ELM groups in the peeling group*.

	Peeling		*P*
	ELM (24)	Non-ELM (10)	
AL	23.34 ± 0.88	23.68 ± 0.91	0.322
Preoperative MD	442.39 ± 145.77	689.50 ± 103.25	0.000
Preoperative length of ELM defect	1062.91 ± 529.36	1780.90 ± 493.90	0.000
RFHE	12/3	1/4	0.031
Preoperative BCVA	0.86 ± 0.39	1.28 ± 0.43	0.006
Postoperative 1-month BCVA	0.58 ± 0.29	1.01 ± 0.54	0.023
Postoperative 12-month BCVA	0.28 ± 0.18	0.86 ± 0.55	0.000
**Postoperative 1-month**			
Hyperreflective change of the inner retina	8/14	10/0	0.001
ELM reconstruction	11/11	0/10	0.006
subretinal cavity	14/8	0/10	0.001
**Postoperative 12-month**			
Hyperreflective change of the inner retina	3/21	8/2	0.000
ELM reconstruction	24/0	0/10	0.000
EZ reconstruction	7/17	0/10	0.078
subretinal cavity	18/6	0/10	0.000

*The power of the chi-squared test is 83%.

### Peeling group: Correlation analysis of the postoperative 12-month ELM reconstruction

The postoperative 12-month ELM reconstruction was closely related to the preoperative MD (P = 0.000, R = –0.656), preoperative ELM defect size (*P* = 0.001, *R* = −0.548), preoperative BCVA (*P* = 0.010, *R* = −0.440), intraoperative RFHEs (*P* = 0.013, *R* = 0.545), and hyperreflective changes in the inner retina (*P* = 0.000, *R* = −0.566) (Figure 4).

## Discussion

In this longitudinal study, the inverted ILM flap improved the postoperative visual acuities of patients with macula holes. However, the postoperative hole closure rates, visual acuities, and ELM reconstruction rates after inverted ILM flap and ILM peeling were not different. There was no difference between the groups based on different MDs. We observed the anatomical reconstruction of the iMH in the flap and peeling groups, respectively. The patients with ILM flaps and hyperreflective changes of the inner retina 1 month postoperatively had more difficult ELM reconstruction 12 months postoperatively. In the peeling group, patients with intraoperative RFHEs and hyperreflective changes of the inner retina also had more difficult ELM reconstruction 12 months after surgery. We speculated that postoperative ELM reconstruction was related to ILM residues in iMHs.

This study showed that the flap and peeling groups could achieve 100% postoperative hole closure rates and improved postoperative visual function, but there was no difference in anatomical morphology and visual function between the flap and peeling groups with different MDs. This was contrary to previous findings (8–10). Since Michalewska reported the inverted ILM flap in 2014, researchers have generally believed that it can improve hole closure rates and visual function in patients with refractory MH (7, 17), but most previous studies involved few cases and did not assess postoperative ELM reconstruction. Recent studies have reported results similar to those of our study (10, 16, 18). Yan (16) suggested that there were no differences between the closure rates and visual acuities after the inverted ILM flap and ILM peeling for iMH. Hasegawa (19) believed that the inverted ILM flap improved the closure rate of large iMHs but not visual function compared with ILM peeling. The inverted ILM flap provides a scaffold for the proliferation and migration of Muller cells and this may account for these findings. However, the ILM flap inevitably fills the hole, and this hinders the rearrangement of the retinal structure and improvement of postoperative visual acuity (16).

The integrities of the EZ and ELM after MH surgery are important indicators. The ELM closure rates at 12 months postoperatively for the flap and peeling groups were not different. Therefore, we further divided the two groups into the ELM and non-ELM groups based on the presence or absence of postoperative ELM reconstruction to observe the differences in the postoperative anatomical reconstruction procedures and the cause of ELM reconstruction.

The Flap group was further divided into two subgroups based on the postoperative 12-month ELM reconstruction. The study showed a higher preoperative MD, more postoperative 1-month hyperreflective changes in the inner retina, and more ILM flaps in the non-ELM group than in the ELM group. The ELM reconstruction was closely related to the preoperative MD, presence of an ILM flap, and hyperreflective changes of the inner retina at 1 month postoperatively. The mechanism of macular hole closure is incompletely understood. Bringmann (20) reported that the closure of iMH was caused by the movement of Muller cells. Muller cells moved toward the fovea to form an accumulation of intermediate reflective tissue and enveloped the photoreceptor cell somata, promoting the centripetal bridging of the ELM. However, the ILM flap was still visible in the non-ELM group at 1 month after surgery in this study, suggesting the co-existence of the ILM and Muller cells in the fovea during the postoperative iMH closure. If these tissues are connected to the RPE, the collagen fibers of the ILM do not degenerate easily, and they form the postoperative hyperreflective changes of the inner retina, which can hinder the centripetal rearrangement of photoreceptors and ELM reconstruction more difficult. These findings showed that the ILM flap did not contribute to the ELM reconstruction of iMH.

The postoperative reconstruction in the peeling group was also observed. The non-ELM groups showed higher preoperative MDs, more intraoperative non-RFHEs, and more postoperative 1-month hyperreflective changes in the inner retina than the ELM group, and the ELM reconstruction was closely related to the preoperative MD, intraoperative RFHEs, and hyperreflective changes in the inner retina. This was consistent with the results of Kumar (21). Previous research reported that RF was similar to the inverted ILM flap mechanism (17); the RFs provided a bridge for the centripetal movement of Muller cells. However, Makoto (22) observed that postoperative BCVA was worse in patients with iMH and RFs than those without them. This may be related to the composition of RF. Son (23) collected some RFs after ILM peeling and found that they only contained RPE cells; however, Compera (24) and Kenawy (25) reported that RFs originated from vitreous cells and Muller cells after histological studies. In the patients in the ELM group, intraoperative RFs often appeared at the edge of the hole, and we speculated they were residual tissues after ILM peeling comprising ILM and retinal tissue. If the ILM was peeled off 360 degrees centripetally and over the MH edge during ILM peeling, the ILM at the edge of the hole would be completely peeled off; the RF residues at the edge of the hole would be the only remaining retinal tissue, which is conducive to postoperative recovery. However, this needs to be further confirmed by more accurate histological studies.

In this study, we speculated that postoperative ELM reconstruction is associated with ILM residues. The postoperative ELM closure rate may be significantly improved if ILM residue is reduced regardless of treatment with an ILM flap or ILM peeling. However, this still requires further histological studies.

This study has some limitations. First, it involved a few cases. Tables 3, 5 have smaller power because their samples are small, but the follow-up period for this study was long, and the data were relatively rare, which can provide clinical guiding significance to readers, and we look forward to the results of large samples in the future. Second, this was a retrospective study. Third, in this study, all patients in the peeling group were treated with centripetal ILM peeling, but iOCT detected RFs in 58.8% of the patients, which was similar to the 54.0–67.0% reported in a previous study. Among the patients with RFs, only 65.0% of RFs were located at the edge of the hole. Therefore, the approach to obtaining satisfactory RFs requires further discussion.

In conclusion, we found no difference between the postoperative BCVAs and rates of ELM reconstruction of the flap and peeling groups subcategorized by MDs. The inverted ILM flap treatment is not recommended for patients with iMHs, as the flap may hinder the normal postoperative reconstruction of iMHs. However, the presence of RFHEs after traditional ILM peeling is closely related to postoperative ELM reconstruction. This may suggest that complete ILM peeling is effective for treating iMHs with different MDs.

## Data availability statement

The raw data supporting the conclusions of this article will be made available by the authors, without undue reservation.

## Ethics statement

The studies involving human participants were reviewed and approved by Institutional Review Board of Wenzhou Medical University. The patients/participants provided their written informed consent to participate in this study.

## Author contributions

LS, JM, and YC contributed to the conception of the study. YC, YX, and XY performed the experiment. YX, JY, and CW contributed significantly to analysis and manuscript preparation. YX and CW performed the data analyses and wrote the manuscript. ZZ helped perform the analysis with constructive discussions. All authors contributed to the article and approved the submitted version.
